# Acute intraperitoneal rupture of hydatid cysts: a surgical experience with 14 cases

**DOI:** 10.1186/1749-7922-8-28

**Published:** 2013-07-26

**Authors:** Ouadii Mouaqit, Abdelaziz Hibatallah, Abdelmalek Oussaden, Khalid Maazaz, Khalid Ait Taleb

**Affiliations:** 1 Surgery Department, Hassan II Hospital, University Sidi Mohamed Ben Abdellah, BP 1893; Km 2.200, Sidi Harazem Road, Fez 30000, Morocco; 2 46, Avenue Ibn el Khatib, Immeuble 46, Lotissement Ghazali, Quartier elAzhar, Fes, Morocco

**Keywords:** Echinococcosis, Abdomen, Peritonitis, Surgery

## Abstract

**Introduction:**

Hydatidosis is endemic in the Mediterranean region including morrocco, the Middle and Far East, Australia, New Zealand, and South America—all areas where animal husbandry is common. Rupture into the abdominal cavity is a rare but serious complication of hydatid disease. The cysts may be ruptured after a trauma, or spontaneously as a result of increased intracystic pressure. Rupture of the hydatid cyst requires emergency surgical intervention.

**Methods:**

Fourteen patients received surgical treatment for intraperitoneal rupture of the cysts over a period of 5 years. Age, gender, time to surgery from the onset of the symptoms, laboratory findings, diagnostic procedures, surgical treatment modalities, in-hospital stay, morbidity, mortality and recurrence were evaluated retrospectively.

**Results:**

Eight of the patients were men and six were women. All of the patients had signs of peritoneal irritation. One patient (7,14%) had a history of blunt abdominal trauma. Ultrasonography scans revealed intra-abdominal fluid in all cases, intraperitoneal multiple cysts in 11 cases and heterogeneous cavity or cystic structures in the liver in 12 cases. Computed tomography showed multiple cystic lesions in the liver and peritoneum with intra-abdominal free fluid. The ruptured cysts were located in the right lobe of the liver in seven patients, in the left lobe in six patients and in both lobes in one patients. Procedures to fill the cystic cavities were applied after removal of the intraperitoneal fluid. Partial pericystectomy and drainage was the most frequent surgical procedure. No patients died in the early postoperative period. A total of seven morbidities developed in six patients (35.3%). Median hospital stay was 08 days and median follow-up was 12 months. Intra-abdominal recurrence occurred in one case (7.7%).

**Conclusions:**

Rupture of hydatid cysts into the peritoneal cavity, although rare, presents a challenge for surgeons. This pathology should be included in the differential diagnosis of acute abdomen in endemic areas. The operative procedures, either radical or conservative, should be based on the patient’s condition, the regional characteristics, and the surgeon’s experience. The morbidity and mortality rates of surgical interventions for ruptured hydatid cysts are higher than the rates for elective uncomplicated cases.

## Introduction

Human hydatid disease usually occurs by infestation with Echinococcus granulosus and less frequently with Echinococcus multilocularis [1]. Although reported from several countries, the disease is endemic in the Mediterranean region, Far East, South America, and Middle East [2,3]. In humans, 50% to 75% of hydatid cysts occur in the liver, 25% are found in the lungs, and 5% to 10% are distributed along the arterial system [4]. Complications of hepatic hydatid cysts are rupture and secondary bacterial infection [4-6]. Primary peritoneal hydatidosis is rare (2%), and the mechanism of this infection is unknown [3]. The cyst may be ruptured after a trauma, or spontaneously as a result of increased intracystic pressure. Superficially located cysts, large cysts, and viable cysts with high pressure are especially prone to rupture into body cavities such as the pleural space and peritoneal cavity, or they may drain into the biliary tract or the gastrointestinal system. The main diagnostic methods are ultrasonography (US) and computed tomography (CT). Presentation is usually dramatic with acute abdominal signs, such as guarding, rebound, and tenderness, are generally present. This complication should be included in the differential diagnosis of acute abdomen, especially in the endemic areas. In patients with peritoneal perforation, specific management has not been evaluated sufficiently, and no clear guidelines are available. The main treatment modalities for uncomplicated cases are also valid for complicated ones, such as peritoneal perforation. Rupture of a hydatid cyst requires emergency surgical intervention [7]. In this study we evaluated 14 hepatic hydatid disease cases with rupture into the peritoneum with regard to surgical treatment modalities and postoperative morbidity and mortality rates.

## Materials and methods

Between January 2008 and December 2012, 306 patients with hydatid disease underwent surgery in our clinic. Fourteen hepatic disease of those patients received surgical treatment for intraperitoneal rupture of the cysts. Patient age and sex, initial complaints, physical findings, laboratory data, imaging results, surgical procedures, reasons for perforation, morbidity, and mortality were evaluated. The preoperative evaluation included blood tests, chest radiography, abdominal ultrasound US, and abdominal computed tomography (CT). All of the patients received epinephrine to prevent allergic reactions preoperatively. Laparotomy through a wide median incision was performed. Besides managing peritoneal dissemination, definitive treatment of intact cysts, if present, was applied. After evacuation, the cyst cavity was irrigated with 3% hypertonic saline or hydrogen peroxide for 10 to 15 min, and the peritoneum was lavaged with 3% hypertonic saline. Any orifice of bile ducts observed on the inner surface of the cavity was sutured with nonabsorbable sutures. Next, a surgical procedure such as partial pericystectomy (PP) and capitonnage, PP and omentoplasty, or PP and drainage was performed. Nearly 2 liters of irrigation fluid was used per patient. Multiple drains were placed before the abdomen was closed in each case. Albendazole treatment (10 mg/kg per day) was given to all of the patients for 12 months postoperatively to prevent recurrence. The patients were seen periodically in the postoperative period, every 3 months during the first postoperative year, every 6 months during the second year, and annually thereafter. Ultrasonography, CT, and indirect hem agglutination tests were performed to detect any recurrence. The study was performed according to the declaration of Helsinki and approved by the Local Ethical Committee.

## Results

**Figure 1:**
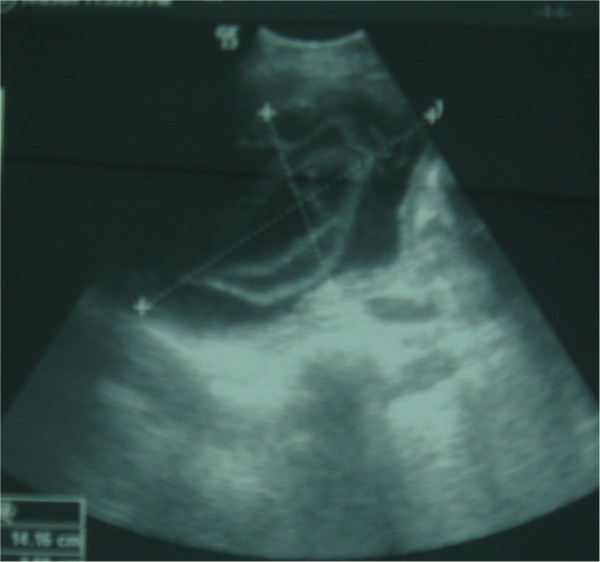
US images showing ruptured hydatid cysts of the liver.

**Figure 2:**
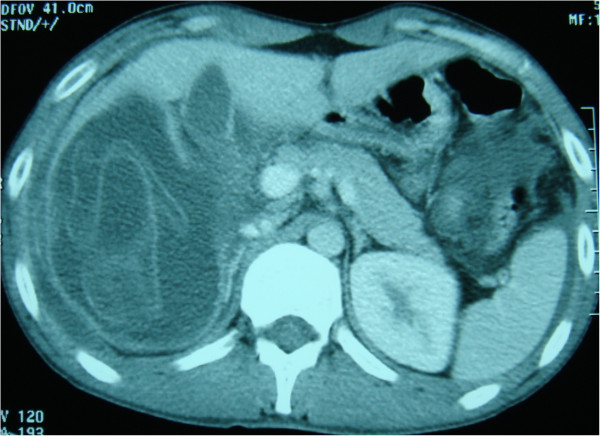
Axial contrast enhanced computed tomography images demonstrate ruptured hydatid lesion within right liver lobe.

**Figure 3:**
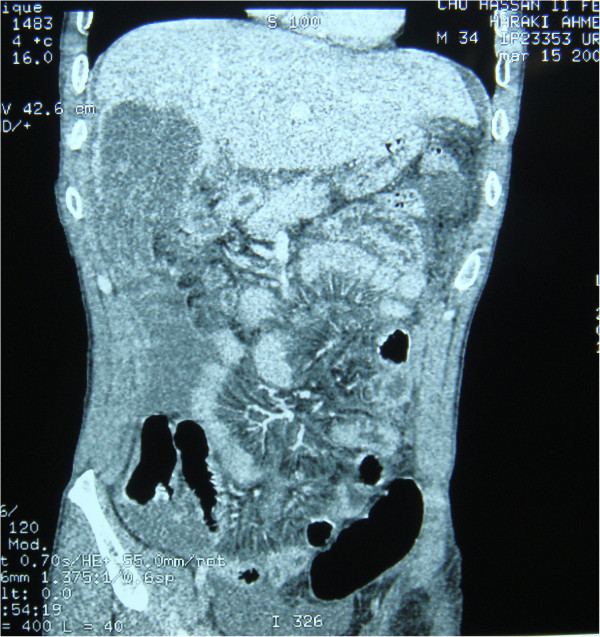
Coronal contrast enhanced computed tomography images demonstrate ruptured hydatid lesion within right liver lobe with perihepatic free fluid.

**Figure 4:**
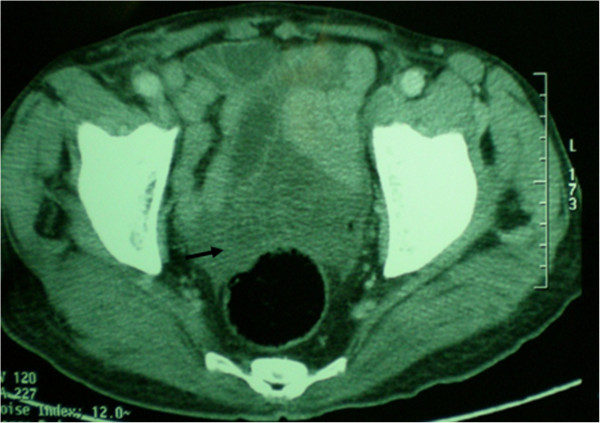
Axial contrast enhanced computed tomography images demonstrate ruptured hydatid lesion with free serous pelvic fluid.

Eight of the patients were men and six were women. Mean age was 39.5 years (range: 20–76 years) (Table 1). All of the patients had signs of peritoneal irritation such as extensive tenderness and guarding. one patients had a history of blunt abdominal trauma (minor abdominal trauma) but 13 patients did not describe any trauma. two patients did not have any complaints prior to the rupture of the cysts, whereas twelve had nonspecific abdominal pain. No patient had previous diagnosis of hydatid disease. Eight patients had fever, 11 had elevated white blood cell count. Ultrasound scans were obtained for all of the patients. Computed tomography scans was available for 13 patients. Ultrasound scans revealed intra-abdominal fluid in all cases, Intraperitoneal multiple cysts in 11 cases (sensitivity = 78.6%) (Figure 1) and heterogeneous cavity or cystic structures in the liver in 12 cases (sensitivity = 85.7%). Both CT showed multiple cystic lesions in the liver and peritoneum with intra-abdominal free fluid (Figures 2, 3, 4). Extensively dilated biliary ducts due to intrabiliary rupture were seen in one case. The ruptured cysts were located in the right lobe of the liver in seven patients, in the left lobe in six patients and in both lobes in one patients. Cysts were single in 8 cases (78%) and multiple in 6 cases (22%). The cysts were infected in four patients (28,6). In both cases, cystic infection was determined incidentally during the operation.

**Table 1:** Patient and cyst characteristics

	**Number of patients (%)**
**Age**	
*mean*	39,5 ± 18,5
*median*	30 (20-70)
**Sex**	
*Male*	8(57,2)
*Female*	6(42,8)
**Previous hydatid disease surgery**	
*Yes*	0
*no*	14(100)
**No. of cysts**	
1	7(50,0)
2	5(35,7)
3	1(7,1)
4	1(7,1)
**Cyst diameter (cm)**	
1-5	0
6-10	5(35,7)
>10	9(64,3)
**Position**	
*Superficial*	11(78,6)
*Deep*	3(21,4)
**Bile content**	
*Positive*	6(42,8)
*Negative*	8(57,2)
**Cyst infection**	
*Positive*	4(28,6)
*Negative*	10(71,4)

**Figure 5:**
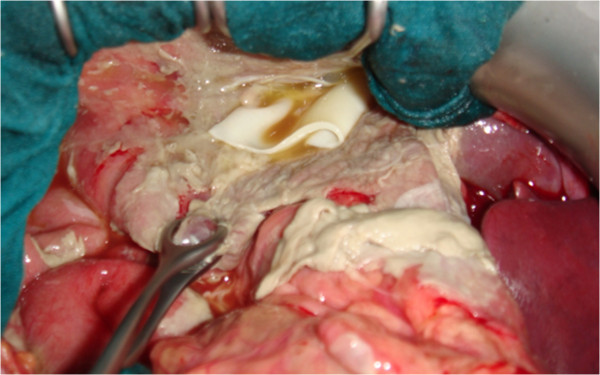
Intraoperative appearance of a cyst in the abdomen.

Besides the ruptured cyst, intact hepatic hydatid cysts were present in six patients and were definitively treated during the surgery. All patients underwent surgery within the first 48 hours after presentation (mean 7 hours). One to five liters of hydatid fluid with floating daughter cysts and purulent material was present in the abdomen (Figure 5). Partial pericystectomy and drainage was the most frequent surgical procedure. In two patient, there was direct communication between the cyst and the gallbladder, and cholecystectomy was performed. Procedures to fill the cystic cavities were applied after removal of the intraperitoneal fluid. Unroofing the cyst, capitonnage and external drainage in all patients, omentoplasty in two patients, were the methods used to manage the cysts. Four patients had two or more of these procedures. No patients died in the early postoperative period. A total of seven complications developed in six patients. biliary fistula developed in two patients. Other complications were prolonged ileus, pulmonary infection, and wound infection, one each. Biliary fistula closed spontaneously in tow of the fistula patients. Sites of the primary cysts, surgical procedures, and postoperative morbidities are shown in Table 2.

**Table 2:** **Site of the primary cysts**, **surgical procedures**, **and postoperative morbidities**

	**Number of patients (%)**
**Site**	
*Liver right lobe only*	7(50)
*Liver left lobe only*	6(42,8)
*Liver both lobes only*	1(7,2)
**Surgery**	
*Partial pericystectomy + drainage*	12(85,7)
*Pericystectomy + drainage*	2(14,3)
*capitonnage*	2(14,3)
*omentoplasty*	2(14,3)
**Morbidity**	
*Total complications*	7(50%)
*Cavitary abscess*	1(7,2%)
*Biliary fistula*	2(14,3)
*Prolonged ileus*	1(7,2%)
*Pulmonary infection*	1(7,2%)
*Eventration*	1(7,2%)
*Wound infection*	1(7,2%)

Median hospital stay was 08 days (range: 6–16 days) and median follow-up was 12 months (1–36 months). Recurrence developed in one patients (7,1%), and these patients underwent additional surgery for this reason.

## Discussion

Infection with echinococcal organisms is the most common cause of liver cysts worldwide [8]. Dogs are the definitive hosts; whereas domestic ruminants (sheep, cattle) and,human are intermediate hosts. Human become hosts accidentally by ingestion of contaminated foods, then ovules of *E*. granulosus are released within duodenum and embryos are. Rupture of a hydatid cyst into the abdominal cavity is a rare complication of the hydatid disease and causes serious problems and severe, life-threatening complications, including anaphylaxis. However, healed cases without anaphylaxis have been reported in the literature as have fatal cases with rupture of the cyst into the peritoneum [7,9,10]. According to Lewall and McCorkell [11], there are 3 types of cyst rupture: contained, communicating, and direct. Various incidence rates of direct rupture have been reported. While Sozuer et al. [12] reported a rate of 8.6%, Beyrouti et al. [7] reported an incidence rate of 1.75%. Rupture can occur spontaneously or following a trauma. The risk of rupture is reported to increase with the increased size of the cyst and intracystic pressure [13]. The main predisposing factors for cyst perforation are young age and superficial localization. Abdominal pain, nausea and vomiting, and urticaria are the most common symptoms [1,3,10]. Allergic reactions may be seen in 25% of the cases. Some authors reported that allergic symptoms occurred in 16.7% to 25.0% of study patients with ruptured hydatid cysts [11,14,15]. Fatal anaphylaxis after cyst rupture has been described [16]. Ultrasound and CT scan may be helpful for defining the cysts with detached membrane and the presence of intraabdominal fluid. Ultrasonography and CT have been reported to be the main diagnostic methods, with 85% and 100% sensitivity, respectively, in identifying hydatid cyst rupture [14,17]. CT yields the most information regarding the position and extent of intra abdominal hydatid disease and demonstrates exogenous cysts. Locating exogenous liver cysts and cysts in other organs is not always possible during surgery. With CT evaluation, more effective interventions can be performed and the incidence of recurrence decreased. the risk factors for cyst perforation were young age, cyst diameter of > 10 cm, and superficial localization [4]. Immediate medical treatment against allergic reactions should be initiated, and emergency surgery should be performed after diagnosing rupture of hydatid cysts. The goal of the surgical treatment is to prevent complications, to eliminate local disease, and to minimize morbidity, mortality, and recurrence rates [7,12]. All of the techniques applied during liver hydatidosis surgery have minor or major disadvantages and are associated with various postoperative complications. The choice of a radical versus a conservative approach is controversial [3,18]. Surgical treatment of the primary cyst should be the aim if the general condition of the patient allows. Pericystectomy and hepatectomy are rarely applied in cases of complicated hydatid cysts, but conservative surgical methods such as external drainage, unroofing, and cavity filling are frequently used [19]. In the study of Gunay et al. [14], only patients who were fit and could tolerate a radical procedure underwent such surgical procedures. Generally, conservative methods are favored in endemic areas, and radical surgery is preferred outside the endemic area. We performed conservative techniques in most cases. Laparoscopic methods and percutaneous drainage of the hydatid cysts has gained interest during the last decade [20,21]. However, we could not find any reports on their use for ruptured cases. We believe that these techniques presently have no place in the management of ruptured hydatid cysts with peritoneal spillage. After intervention for a perforated cyst, the most important step is irrigating the peritoneal cavity with a sufficient amount of scolicidal agents and careful, patient removal of all cystic content. Numerous solutions, such as hypertonic saline solution (15–30%), formalin (2%), silver nitrate (0.5%), povidone-iodine (10%), chlorhexidine (0.05%), and a combination of cetrimide (0.5%) and chlorhexidine (0.4%), have been used as scolicidal agents for the purpose of inactivation [22,23]. we used hypertonic saline solution. Now we use only 3% concentrations. Derici et al. [1] reported that hypertonic saline is not appropriate because it may damage the peritoneal surfaces and may cause hypernatremia, we have not encountered any significant complications with the use of this solution. Additionally, we believe that profuse peritoneal lavage with hypertonic sodium chloride is mandatory for preventing intra abdominal recurrence of hydatid disease. Surgical mortality rates are as much as 3% even after surgery for uncomplicated hydatid cysts [1,3,14,15]. Morbidity has been reported to be 12% to 63% [1,3]. Derici et al., reported four deaths (23.5%) in a series of 17 patients [1]. The literature contains relatively few series of patients with perforated hydatid cysts, and there are more associated organ injuries in these cases, so determining associations between surgical procedures and mortality is difficult. Beyrouti et al., reported four morbidities (23.5%) and two mortalities (11.8%) in a series of 17 patients, and Sozuer et al. reported two complications (10%) but no mortality in 21 patients [7,12]. Deaths were due to septic shock and multiorgan failure. We had no mortality in our study. All patients received albendazole for at least 6 month to reduce recurrence rate. Albendazol treatment is effective for preventing recurrence and secondary hydatidosis, but there is no agreement on the duration of use of the medication for cyst sterilization. The efficacy and safety of albendazole treatment have been demonstrated in various studies [1,3,24]. Recurrence rates were 0% to 13% in other studies [14,25]. Gunay et al. [14] reported no recurrence after a mean follow-up of 30 months. In the studies of Beyrouti et al., and Sozuer and Ackan and Dreci et al., recurrence rates are 6.7% and 14% and 11,1and 7,7 respectively [1,3,7,12]. In the series of Kurt et al., recurrence is reported at 28.6% in seven cases [10]. In our study, there were one cases (7,1%) of recurrent disease.

## Conclusions

Rupture of hydatid cysts into the peritoneal cavity, although rare, still presents a challenge for the surgeon. This pathology should be included in the differential diagnosis of acute abdomen in endemic areas Emergency surgery is the main treatment for intraperitoneal rupture of hydatid cysts, and medical treatment should be given postoperatively. The choice between a radical and a conservative operative procedure should be based on the number, size, and localization of cysts; the relation of cysts to bile ducts and blood vessels; additional organ injuries; and the general condition of the patient. In addition, the morbidity rates of surgical operations are higher among patients with perforated hydatid cysts than in those with noncomplicated cases. It is most important to prevent hydatid infestation.

### Consent

Written informed consent was obtained from the patient for the publication of this report and any accompanying images.

## Abbreviations

CT scan: Computed tomography scan; US: Ultrasonography; PP: Pericystectomy.

## Competing interests

The authors declare that they have no competing interests.

## Authors’ contributions

OM conceived the idea of the study, and also performed and supervised the whole process and operated when required, written and corresponded the manuscript. AH assisted in managing the patients with strict vigilance and helped in the preparation of manuscript. All authors read and approved the final manuscript.
